# An evaluation of serological tests in the diagnosis of bovine brucellosis in naturally infected cattle in KwaZulu-Natal province in South Africa

**DOI:** 10.4102/jsava.v88i0.1381

**Published:** 2017-02-28

**Authors:** Songelwayo L. Chisi, Yoanda Marageni, Prebashni Naidoo, Gloria Zulu, George W. Akol, Henriette van Heerden

**Affiliations:** 1 Department of Agriculture and Rural Development, Allerton Provincial Veterinary Laboratory, South Africa; 2 Centre of Veterinary Excellence, Dohne Agricultural Development Institute, South Africa; 3 Department of Rural Development and Agrarian Reform, Stutterheim, South Africa; 4 Department of Tropical Diseases, University of Pretoria, South Africa

## Abstract

The diagnostic sensitivity (DSe) of the Rose Bengal test (RBT), the complement fixation test (CFT), the serum agglutination test (SAT), the competitive enzyme-linked immunosorbent assay (cELISA) and the indirect ELISA (iELISA) were determined in naturally infected cattle in KwaZulu-Natal province of South Africa with known infectious status from culture (gold standard). Natural brucellosis infection status of animals was determined by culturing and identification of *Brucella abortus* biovar 1 from abomasal fluid, milk, hygroma fluid, lymph nodes or uterine discharges samples. The diagnostic specificity (DSp) of the tests mentioned above was determined using samples from known negative herds. There was no statistically significant difference between the tests in their ability to diagnose brucellosis. The RBT and iELISA had the highest DSe of 95.8%, whereas RBT and CFT had the highest DSp of 100%. In South African laboratories, the RBT and CFT serological tests are used, because of the cost efficacy of CFT when compared to the less labour intensive but more expensive iELISA.

## Introduction

Brucellosis is a worldwide zoonotic disease of economic and public health importance. The disease is caused by an intra-cellular, facultative, Gram-negative bacterium of the genus *Brucella*. Bovine brucellosis is a disease of cattle, usually caused by *Brucella abortus*, less frequently by *Brucella melitensis* and rarely by *Brucella suis* (Bishop, Bosman & Herr 1994). There are eight recognised biovars (bv) of *B. abortus* (Alton et al. 1988; Bishop et al. 1994; Garin-Bastuji et al. 2014). In South Africa, 90% of infections in cattle are because of *B. abortus* bv 1, and 10% are because of *B. abortus* bv 2 (Bishop et al. 1994). The seroprevalence of brucellosis using Rose Bengal test (RBT) and complement fixation test (CFT) in rural cattle in KwaZulu-Natal province is approximately 1.45% according to a survey conducted in rural KwaZulu-Natal communal areas (Hesterberg et al. 2008). An earlier seroprevalence survey (November 1981–August 1982) using RBT and CFT in adult female cattle (mostly of commercial animals) at Cato Ridge abattoir in KwaZulu-Natal indicated 1.5% prevalence (Bishop 1984).

In order for bovine brucellosis surveillance to be effective, reliable diagnostic tests need to be used. Bacterial culture and identification is the gold standard for diagnosis of *B. abortus* (Morgan 1977; Nielsen 2002). However, in some cases, culture yields negative results or is impractical with large herds and huge numbers of animals (World Organisation for Animal Health [OIE] 2012). Furthermore, culture and identification takes at least 2 weeks from sample submission, and *Brucella* cultures need to be handled by trained staff in laboratories with appropriate biosafety. In the absence of bacterial culture, serological tests offer a more practical means of diagnosing brucellosis. However, more than one test is used to confirm brucellosis as no single test absolutely identifies *Brucella*, that is, combinations of culture, serological test(s) and polymerase chain reaction can give a definitive diagnosis.

Serology remains the most practical method available to screen herds and confirm diagnosis. In South Africa, veterinary laboratories have traditionally used RBT, CFT, serum agglutination test (SAT) and milk ring test (MRT) in brucellosis diagnosis. These tests use a whole-cell antigen as the diagnostic reagent (Alton et al. 1988; Van et al. 1984). RBT and CFT are used in combination to confirm bovine brucellosis in many countries (Nielsen 2002; OIE 2012) including South Africa. RBT is used for its higher sensitivity, whereas CFT is used for its higher specificity (Nielsen 2002; OIE 2012). Combination of CFT and SAT serological tests is unreliable because SAT is regarded as an unreliable test and ‘being unsatisfactory for the purpose of international trade’ (OIE 2012). It is essential to identify diagnostic tests that are reliable, specific, cost effective and easy to perform, which will ensure that no uninfected animals are destroyed or that no infected animals remain in a herd because of misclassification.

The primary binding assays include indirect enzyme-linked immunosorbent assay (iELISA), competitive ELISA (cELISA) and the fluorescent polarisation assay (FPA) that employ purified lipopolysaccharide (LPS) or O-antigen as the diagnostic reagent (Nielsen 2002). ELISA tests were developed to be more sensitive and specific alternatives to conventional tests (Gall & Nielsen 2004). The primary binding assay tests are robust and fairly simple to perform with a minimum of equipment, and the iELISA is recommended by the OIE as a suitable screening test (OIE 2012). The conventional serological tests (SAT, RBT and CFT) and iELISA are, however, unable to distinguish between vaccine strain S19 and naturally infected animals (Nielsen et al. 1995). The cELISA was developed to overcome this problem; it is fairly rapid to perform and cross-reacts less with other antigens (or antibodies) than the conventional tests. Further, the cELISA is simpler to perform than the CFT and may readily be standardised by the use of purified S-LPS antigen and monoclonal antibody for competition (Gall & Nielsen 2004; McGiven et al. 2003). One of the major diagnostic problems that result from the use of the O-antigen is false-positive results with bacteria possessing similar O-antigenic side chains of the LPS such as *Yersinia enterocolitica* O: 9 (McGiven et al. 2003; Van et al. 1984).

The current surveillance and control scheme in South Africa uses serological tests consisting of RBT as a screening test and CFT as a confirmatory test. Bleeding and testing of beef and dairy cattle in commercial and communal areas are carried out within defined intervals by state and private veterinarians. Specimens used for the isolation of *Brucella* organisms include milk, hygroma fluid and lochia from live female animals, lymph nodes from carcasses of slaughtered adult animals, foetuses and placenta tissues. Laboratory test results are sent to the state veterinarian, who is responsible for liaising with the animal owner, for further or appropriate actions on the farm, depending on the findings. All animals reacting positive to RBT and CFT are handled in accordance with the applicable control measures (*Animal Diseases Act* 35 of 1984), which include quarantine, branding and slaughter. If all animals in a herd test negative on both RBT and CFT, the herd will be declared free of brucellosis by the state veterinarian and a brucellosis-free certificate will be issued to dairy herds.

In this study, *Brucella* bacterial culture status was determined at Allerton Provincial State Veterinary Laboratory (APVL) from milk, abomasal fluid from aborted foetuses, uterine discharges and hygroma fluid samples from suspected brucellosis-infected cattle from commercial and communal herds in KwaZulu-Natal, as well as lymph nodes (nodes collected from abattoirs that slaughtered known brucellosis seropositive cattle). Serum samples of these cattle with known bacteriological status and *Brucella*-negative status based on brucellosis-free certification by the responsible state veterinarian were obtained and evaluated to determine the diagnostic sensitivity (DSe) and specificity (DSp) of the RBT, CFT, SAT, cELISA and iELISA tests. Furthermore, the serological tests were evaluated on brucellosis-free herd samples.

## Materials and methods

### *Brucella*-negative herds

This study was conducted at APVL, KwaZulu-Natal province, in South Africa. Serum samples were collected from *Brucella*-negative cattle (*n* = 186) from 21 herds that were declared negative by the state veterinarian of the responsible area in KwaZulu-Natal based on negative MRT test in dairy herds or negative CFT test results in beef cattle. Dairy herds are tested every month using the MRT test; the herd is certified *Brucella* negative after 12 consecutive negative tests, and beef cattle herds are certified *Brucella* negative after tests on sera of animals 18 months or older are negative, in which case the herd is re-tested 3–5 years later.

### Naturally infected animals

Brucellosis status using culturing (gold standard, *n* = 46, Table 1) was determined from milk, abomasal fluid from aborted foetuses, uterine discharges and hygroma fluid samples from suspected brucellosis-infected cattle from commercial and communal herds in KwaZulu-Natal submitted to APVL. These samples also included lymph nodes that were collected at abattoirs from brucellosis seropositive animals with high CFT titres (392 or greater CFT IU) that were slaughtered under supervision of a state veterinarian according to the law (Act 35 of 1984). The *Brucella*-positive status of these animals was established through bacterial culture from lymph nodes. The samples used in this study were collected between 2009 and 2012.

**TABLE 1:** The two-by-two contingency table used to arrange data to calculate the diagnostic sensitivity of each serological test using bacterial culture of *Brucella abortus* bv 1 as the gold standard.

Test result	Culture status
-	Positive (infected)
Positive	True positive (a)
Negative	False negative (b)

**Total**	**True positive + false negative (a + b)**

Diagnostic sensitivity = a (a + b)

### Bacteriology

Abomasal fluid, milk, hygroma fluid, uterine discharges and lymph nodes samples were inoculated onto *Brucella* selective medium and blood agar (BA) (Quinn et al. 1994) and incubated at 37 °C (with 6% O_2_, 10% CO_2_ and 84% N_2_). Plates were examined daily for the first 7 days and discarded as negative after 14 days if no growth of colonies was observed. Suspected *Brucella* colonies were stained with modified Ziehl–Nielsen (Stamp) and oxidase, urease and catalase tests were performed (Quinn et al. 1994). Suspected colonies that grew in CO_2_ and tested positive for urease, catalase, oxidase and stained positive for Ziehl–Nielsen were considered positives. Identification of *Brucella* colonies at strain level as indicated by Alton et al. (1988) and OIE (2012) was done at the Agricultural Research Council – Onderstepoort Veterinary Institute (ARC-OVI) reference laboratory at Onderstepoort in South Africa.

### Serology

Serum samples were collected from cattle with known or suspected brucellosis status by state veterinarians, animal health technicians (AHT) or private veterinarians in KwaZulu-Natal province and sent to APVL. Serum samples of naturally infected animals were tested for *B. abortus* antibodies using RBT, CFT and SAT as part of the brucellosis surveillance programme using recommended cut-off points by the Department of Agriculture, Forestry and Fisheries, South Africa (Department of Agriculture, Forestry and Fisheries 2013). Serum samples from certified *Brucella*-negative herds were similarly tested. In this study, samples were further tested using iELISA and cELISA.

#### Rose Bengal test

Sera from bovine were tested for antibodies against *Brucella* antigen using RBT. This was performed at APVL as described by Alton et al. (1988). The whole-cell antigen and positive control were procured from Ondersterpoort Biological Products (OBP), South Africa.

#### Complement fixation test

This test was performed according to a described procedure (Department of Agriculture, Forestry and Fisheries 2013) that tests for antibodies against *B. abortus* in bovine serum. Positive, anti-complementary and negative controls were included in each test as controls. If haemolysis was present, the sample was recorded as negative, and a lack of haemolysis, indicated by the formation of a red button at the centre of the well, was recorded as positive. Samples from correctly vaccinated herds with a titre of 60 CFT IU or greater were considered positive.

#### Serum agglutination test

The SAT was performed according to the procedure described previously (Alton et al. 1988; Herr, Te Brugge & Guiney 1982). Those samples with titres greater than 161 SAT IU were considered positive. Positive and negative controls were included.

#### Indirect enzyme-linked immunosorbent assay

The bovine brucellosis iELISA kit (Institut Pouquirer) was used according to the manufacturer’s instruction. The sample results were calculated as positive percentage relative to the strong positive control serum (S/P) using the formula:

S/P%=100×[(Sample OD−negative control OD)/(Mean OD of positive control−negative control)]. [Eqn 1]

A sample was considered positive if the S/P value was greater than 120%.

#### Competitive enzyme-linked immunosorbent assay

The *Brucella* cELISA kit (Svanovir) was used according to the manufacturer’s instruction. Reactivity was calculated as percentage inhibition (PI), using the formula:

PI=100−[(Mean OD of sample/Mean OD of conjugate control)×100]. [Eqn 2]

A sample was considered positive if the PI value was greater than 30%.

### Statistical analysis

The two-by-two contingency table (Table 1) was used to arrange data so that the DSe was calculated to determine the performance of each of the serological tests using bacterial culture as the gold standard. Chi-square analysis was used to assess if there were any statistically significant differences amongst the various tests in diagnosing brucellosis in this study. DSp was estimated in *Brucella*-free herds by testing animal populations known to be free of the disease, that is, DSp = animals testing negative/total number of animals tested (Table 2). The diagnostic performance of the tests was also assessed by computing the DSe when tests are used in series or in parallel. This was done by evaluating test combinations involving RBT and iELISA tests (Tables 3 and 4). In series testing, a sample was considered positive if both tests were positive. DSe was then computed. In case of parallel testing, a sample was considered positive if it tested positive to one or both tests.

**TABLE 2:** Information regarding diagnostic specificity of *Brucella*-free herds for serological tests namely Rose Bengal test, complement fixation test, serum agglutination test, competitive enzyme-linked immunosorbent assay and indirect enzyme-linked immunosorbent assay.

Serological tests	RBT	CFT	SAT	cELISA	iELISA
Total number of sera tested	186	186	186	180	186
Number of negative samples	186	186	180	171	172
Number of positive samples	0	0	6	9	8
Diagnostic specificity (DSp %)	100	100	96.8	95.0	92.5
Confidence interval (at 95%)	-	-	93.6–98.8	90.7–97.7	88.3–96.2

DSp, diagnostic specificity; RBT, Rose Bengal test; CFT, complement fixation test; SAT, serum agglutination test; cELISA, competitive ELISA; iELISA, indirect ELISA.

**TABLE 3:** Diagnostic sensitivity results for various Rose Bengal test and indirect enzyme-linked immunosorbent assay combinations using series testing.

Combination	DSe
**RBT**	
+RBT/+CFT	91.3 (79.2–97.6)
+RBT/SAT	87.0 (73.7–95.1)
+RBT/+iELISA	93.5 (82.1–98.6)
+RBT/+cELISA	89.1 (76.4–96.4)
**iELISA**	
iELISA/RBT	93.5 (82.1–98.6)
iELISA/CFT	91.3 (79.2–97.6)
iELISA/SAT	87.0 (73.7–95.1)
iELISA/cELISA	89.1 (76.4–96.4)

RBT, Rose Bengal test; CFT, complement fixation test; iELISA, indirect ELISA; cELISA, competitive ELISA; SAT, serum agglutination test; DSe, diagnostic sensitivity.

**TABLE 4:** Diagnostic sensitivity results for various Rose Bengal test and indirect enzyme-linked immunosorbent assay combinations using parallel testing.

Combination	DSe
**RBT**	
+RBT/+CFT or +RBT or +CFT	97.8 (88.5–99.0)
+RBT/SAT or +RBT or +SAT	97.8 (88.5–99.0)
+RBT/+iELISA or +RBT or +iELISA	97.8 (88.5–99.0)
+RBT/+cELISA or +RBT or +cELISA	97.8 (88.5–99.0)
**iELISA**	
+iELISA /+RBT/+ or +RBT or +iELISA	97.8 (88.5–99.0)
+iELISA/+CFT + or +iELISA +CFT	97.8 (88.5–99.0)
+iELISA/+SAT or +iELISA +SAT	87.8 (73.7–95.1)
+iELISA/+cELISA or +iELISA or +cELISA	100.0

RBT, Rose Bengal test; CFT, complement fixation test; iELISA, indirect ELISA; cELISA, competitive ELISA; SAT, serum agglutination test; DSe, diagnostic sensitivity.

## Results

The *Brucella* bacterial culture status from 46 samples from milk, abomasal fluid from aborted foetuses, uterine discharges and hygroma fluid from cattle of commercial and communal herds in KwaZulu-Natal, as well as lymph nodes (nodes collected from abattoirs that slaughtered known brucellosis seropositive cattle), is indicated in Table 5. These isolates came from 21 farms. These 46 *Brucella* cultures were isolated from these 46 samples (abomasal fluid, hygroma fluid, milk and lymph nodes).

**TABLE 5:** Results of brucellosis serological testing performed at Allerton Provincial Veterinary Laboratory.

Sample number†	*Brucella* culture status‡	Sample type§	Serological test¶				

			RBT (Pos/Neg)	CFT (> 49 CFT IU)	SAT (161 SAT IU)	cELISA (30% PI)	iELISA (120%)
B-1	Pos	abofluid	Pos	Pos	pos	pos	pos
B-8	Pos	abofluid	Pos	Pos	pos	pos	pos
B-9	Pos	abofluid	Pos	Pos	pos	pos	pos
B-10	Pos	abofluid	Pos	Pos	pos	pos	pos
B-11	Pos	abofluid	Neg	Pos	pos	pos	pos
B-12	Pos	abofluid	Pos	Pos	pos	pos	neg
B-13	Pos	abofluid	Pos	Pos	pos	pos	pos
BA-1	Pos	abofluid	Pos	Pos	pos	pos	pos
BA-2	Pos	abofluid	Pos	Pos	pos	pos	pos
BA-3	Pos	lymphno	Pos	Pos	pos	pos	pos
BA-4	Pos	lymphno	Pos	Neg	neg	pos	neg
BA-5	Pos	abofluid	Neg	Neg	neg	neg	pos
L-1	Pos	lymphno	Pos	pos	pos	pos	pos
M-1	Pos	hygroma	Pos	pos	pos	pos	pos
ML-1	Pos	lymphno	Pos	pos	pos	pos	pos
ML-2	Pos	lymphno	Pos	pos	pos	pos	pos
ML-3	Pos	lymphno	Pos	pos	pos	pos	pos
ML-4	Pos	lymphno	Pos	pos	pos	pos	pos
MS-1	Pos	lymphno	Pos	pos	pos	pos	pos
MS-2	Pos	lymphno	Pos	pos	pos	pos	pos
MS-3	Pos	lymphno	Pos	pos	pos	pos	pos
MS-4	Pos	lymphno	Pos	pos	pos	pos	pos
MS-5	Pos	lymphno	Pos	pos	pos	pos	pos
MS-6	Pos	lymphno	Pos	pos	pos	pos	pos
MS-7	Pos	lymphno	Pos	pos	pos	pos	pos
MS-8	Pos	lymphno	Pos	pos	pos	pos	pos
MS-9	Pos	lymphno	Pos	pos	pos	pos	pos
N-1	Pos	lymphno	Pos	pos	pos	pos	pos
N-2	Pos	hygroma	Pos	pos	pos	pos	pos
N-3	Pos	hygroma	Pos	pos	neg	pos	pos
N-4	Pos	hygroma	Pos	pos	neg	neg	pos
N-5	Pos	hygroma	Pos	pos	pos	pos	pos
N-6	Pos	hygroma	Pos	pos	pos	pos	pos
N-7	Pos	hygroma	Pos	pos	pos	pos	pos
N-8	Pos	hygroma	Pos	pos	pos	pos	pos
N-9	Pos	hygroma	Pos	pos	pos	neg	pos
N-10	Pos	hygroma	Pos	pos	pos	pos	pos
R-1	Pos	lymphno	Pos	neg	pos	pos	pos
R-2	Pos	lymphno	Pos	pos	neg	pos	pos
S-1	Pos	lymphno	Pos	pos	pos	pos	pos
ST-3	Pos	abofluid	Pos	pos	pos	pos	pos
SVNC-1	Pos	abofluid	Pos	pos	pos	pos	pos
V-1	Pos	abofluid	Pos	pos	pos	pos	pos
V-2	Pos	abofluid	Pos	pos	pos	pos	pos
WG-1	Pos	lymphno	Pos	pos	pos	pos	pos
WG-2	Pos	lymphno	Pos	pos	pos	pos	pos

† , The letters of the samples indicated farms and numbers indicate the amount of samples from each farm and all the farms in Table 1 had abortion history;

‡ , culture status where (pos) indicates *Brucella abortus* isolated;

§ , abofluid: abomasal fluid from aborted foetus, lymphno: lymph node, hygroma: hygroma fluid;

¶ , Serological tests results from serum samples where (pos) indicate seropositive.

Serology using RBT, CFT, iELISA, cELISA and SAT was performed on 46 *Brucella*-positive samples confirmed by *B. abortus* culture (Table 5). The iELISA and RBT had the highest DSe of 95.8% [95% confidence interval (CI): 85.8% – 99.5%] followed by cELISA and CFT that had a DSe of 93.9% (95% CI: 71.1% – 98.7%) whilst SAT had a DSe of 92.0 % (95% CI: 78.6% – 96.6%). Using sera from *Brucella*-free herds, the CFT and RBT had the highest DSp of 100.0%, followed by SAT and cELISA with a DSp of 96.8% (95% CI: 93.6% – 98.8%) and 95.0% (95% CI: 90.7% – 97.7%) whilst the iELISA had the lowest DSp of 92.5% (88.3% – 96.2%). The DSe and DSp total for each test was calculated and established RBT and CFT (195.8 and 193.9, respectively) as the most sensitive and specific tests (Table 6). The chi-square analysis was done, and a *P*-value of 0.9 at 95% CI was calculated using RBT, SAT, CFT, cELISA and iELISA indicating that there was no significant statistical difference between the tests in diagnosing *B. abortus*. The RBT and iELISA combination had the highest DSe, that is, 93.5% (95% CI: 82.1% – 98.6%) when tested in series (Table 5), whereas the iELISA and cELISA combination had the highest DSe, that is, 100% when parallel testing was used to assess performance.

**TABLE 6:** Diagnostic sensitivity and diagnostic specificity results for serological tests including Rose Bengal test, complement fixation test, serum agglutination test, competitive enzyme-linked immunosorbent assay, indirect enzyme-linked immunosorbent assay with known *Brucella abortus* bv 1 bacterial culture status.

Tests	Diagnostic sensitivity				DSe + DSp (Performance index)

	DSe %	CI at 95%†	DSp %	(CI at 95%)‡	
RBT	95.8	85.8–99.5	100	-	195.8
CFT	93.9	81.1–98.7	100	-	193.9
iELISA	95.8	85.8–99.5	92.5	88.3–96.2	188.3
cELISA	93.9	83.1–98.7	95.0	90.7–97.7	188.9
SAT	90.2	78.6–96.7	96.8	93.6–98.8	166.4

Confidence interval (CI) at 95%.

RBT, Rose Bengal test; CFT, complement fixation test; iELISA, indirect ELISA; cELISA, competitive ELISA; SAT, serum agglutination test; DSe, diagnostic sensitivity; DSp, diagnostic specificity.

† , Diagnostic sensitivity of samples with known bacterial culture results (*n* = 46);

‡ , diagnostic specificity of *Brucella*-free herds with *n* = 186 (Table 4).

## Ethical considerations

The results of this research will be made available to the veterinary and farming community of KwaZulu-Natal.

## Discussion

This study indicated that iELISA and RBT had the highest DSe (Table 3), whereas RBT and CFT had the highest diagnostic specificity (DSp) (Tables 3 and 4) of all the serological tests for bovine brucellosis in naturally infected cattle in KwaZulu-Natal province. Currently, South African brucellosis surveillance strategy uses both RBT and CFT to establish brucellosis status of animals and herds. Literature indicates that primary binding tests have a better performance than the currently used RBT and CFT (Gall & Nielsen 2004; McGiven et al. 2003; Muma et al. 2007; Paweska et al. 2002). However, most of these studies investigated the performance of serological test without a gold reference test to classify animals as *B. abortus* infected and non-infected.

When comparing values from this study with other studies (Abernethy et al. 2012; Gall & Nielsen 2004; McGiven et al. 2003; Muma et al. 2007; Paweska et al. 2002), the sensitivity and specificity values differ, and most of these studies did not use gold standard reference samples (samples with known infection status using culture). The variation in test results is also clear from the meta-analysis study that investigated the performance of serological tests based on sensitivity and specificity values (Gall & Nielsen 2004). The reasons for variation in sensitivity and specificity of the serological tests are factors like the infection status of the sample being unknown, transport and storage conditions of sample from collection to the laboratory, as well as factors not disclosed by owners (i.e. vaccination status). Even if infectious status based on culture is available, there are still unknown variables that influence the results. Animals BA-4 and BA-5 were infected with *B. abortus* (Table 5), but most of the serological tests were negative, which might be attributed to the quality of the sample. No single test identified all of the infected cattle. This was also reported by Abernethy et al. (2012) using six serological tests on herds selected to alleged exposure to *B. abortus* with cases defined by culture or test agreement.

Series testing of sera using RBT and CFT is practiced at APVL, and the possibility exists that a positive animal is misdiagnosed. With the RBT screening test, negative results can be obtained because of prozone as was the case with sample B-11. The prozone phenomenon in humans was assessed to be present in patients with past infection (Salih et al. 2007) and with SAT and Coombs tests in samples with too low dilutions (Karsen et al. 2011). This is a possible explanation for sample B-11 as most of the other animals on this farm were positive. Follow-up testing is done in positive herds using RBT and CFT (Department of Agriculture, Forestry and Fisheries 2013). The DSe of CFT and RBT was 93.9% and 95.8%, respectively, in this study. The CFT was slightly higher than the 83% and 89% found by Paweska et al. (2002) and Gall and Nielsen (2004), respectively. Paweska et al. (2002) used a cut-off of 20 CFT IU and did not verify infection with culture as opposed to > 49 CFT IU cut-off in this study with known infection status.

Findings from a Canadian study using infected cattle confirmed by culture found the DSe for SAT to be 93.3% at a cut-off of 80 SAT IU (Dohoo et al. 1986), which is very similar to the 90.2% of this study. The lower DSe value is most probably because of the higher cut-off of 161 SAT IU. The SAT test indicated B-2 and B-5 as false positives. This test detects IgM, the main immuglobulin produced after vaccination, or it can be attributed to an early infection also as IgM is the main immunoglobulin produced in early infection (Nielsen 2002). Interpretation of both SAT and RBT test results is subjective (Weiner et al. 2010). Samples N-3, N-4 and R-2 were classified as false negatives according to SAT results. This problem is encountered in chronically infected herds which produce IgG as opposed to IgM (Godfroid, Nielsen & Saegerman 2010).

A number of studies have reported that the primary binding assays, that is, cELISA and iELISA, have higher DSe and DSp, therefore deeming these assays to perform better than the conventional tests (Gall & Nielsen 2004; McGiven et al. 2003; Muma et al. 2007; Paweska et al. 2002). ELISAs have been reported to be easy to perform, less labour intensive and reliable (Perrett et al. 2010). However, in this study, the researcher encountered difficulties with the cELISA. Furthermore, the cELISA controls were out of range and the test had to be repeated to obtain reliable results on a number of runs. This could be attributed to the inexperience of the researcher with this test or the test kit was sensitive to temperature fluctuations. These technical difficulties were not experienced using the iELISA test kit. The quality of the samples at the time of testing might explain the seronegative results of BA-4 and BA-5 for iELISA and cELISA, respectively. In our study, the iELISA had the joint highest DSe of 95.8% with the RBT.

## Conclusion

The aim of this study was to evaluate the performance of the CFT, cELISA, iELISA, RBT and SAT in diagnosing bovine brucellosis in naturally infected cattle in KwaZulu Natal province of South Africa. No statistically significant differences in the performance of RBT, SAT, CFT, cELISA and iELISA in the diagnosis of bovine brucellosis in naturally infected cattle in KwaZulu-Natal were found in this study using chi-square analysis with a *p*-value of 0.9 at 95% CI. The iELISA and RBT had the highest DSe, whereas RBT and CFT had the highest DSp for bovine brucellosis in naturally infected cattle in KwaZulu-Natal province of all the serological tests. This can be observed with the series testing of RBT and CFT where four *B. abortus*-infected animals (B-11, BA-4, BA-5 and R-1) would have been misdiagnosed as negative. The series testing using RBT and iELISA is slightly more sensitive where three *B. abortus*-infected animals (B-11, BA-4 and BA-5) would have been misdiagnosed as negative (Table 1) from samples used in this study. Results of this study indicate that the performance of traditional tests used to diagnose bovine brucellosis in South Africa, namely RBT and CFT, is similar to primary binding assays (iELISA and to a lesser extent cELISA) in diagnosing naturally infected cattle. RBT and CFT performed better than the cELISA and iELISA in *Brucella*-free herds with a DSp of 100%. However, the iELISA and cELISA combination had the highest DSe, that is, 100% when tested in parallel (Table 4). The cost of the ELISA kits prohibits the use in developing countries, a fact not taken into account by McGiven et al. (2003) and others that indicate ELISA as an automated, rapid, easy test that renders itself easily to quality control measures. Because of the cost, RBT and CFT combination remains the best to detect *Brucella* infections at the APVL in KwaZulu-Natal province. Further investigations to establish optimum cutoff points for the CFT using ROC analysis are recommended.
